# Life at the academic coalface: validation of a holistic academic workload estimation tool

**DOI:** 10.1007/s10734-022-00912-x

**Published:** 2022-08-13

**Authors:** John Kenny, Andrew Edward Fluck

**Affiliations:** Faculty of Education, University of Tasmania, Launceston, Australia

**Keywords:** Academic workload, Academic performance, Estimating academic work

## Abstract

This paper reports on research exploring the academic workload and performance practices of Australian universities. This research has identified a suite of activities associated with teaching, research and service, each with an associated time value (allocation). This led to the development of the academic workload estimation tool (AWET). In 2020, to validate the findings, we contacted academics willing to participate further and conducted interviews. We used the AWET to estimate workload for each individual for the previous year and compared it to the workload allocated according to their institutional workload model. Discrepancies were then discussed to ascertain to what extent the AWET was able to capture their work. In general, the participants thought the AWET provided a more realistic estimate of their actual work and highlighted how much is underestimated or unaccounted for by the workload models used within their institutions. It also showed how academic performance policies, focussed primarily on research output, disadvantaged many individuals because they ignored or minimised many scholarly, teaching and service-related tasks inherent in the academic role. Overall, the findings showed the AWET was a useful tool to discuss academic work and assisted them to better capture the complexity and extent of what they did. We offer the AWET as a validated approach for academics to estimate their workload in a holistic and transparent manner. We suggest its implementation institution-wide, along with an aligned performance policy, will facilitate negotiation of reasonable performance expectations. This will rebuild trust in the processes and improve a university’s effectiveness.

## Introduction

Ernest Boyer’s (1990) four “scholarships” of discovery, integration, application and teaching described the interrelated and scholarly nature of academic work. He emphasised the importance of pure and applied research, community engagement, service and teaching, and recognised that each potentially contributes to the advancement of knowledge in a field.

Academics today are still concerned with these scholarships, but the context in which they work has undergone profound change. For example, as an export industry, Australian universities contributed $41 billion to the Australian economy in 2018 (Universities Australia, 2020, p. 3). Reduced government funding and increased external accountability have led to a shift in university governance from collegial to more hierarchical managerial forms (Henkel, 2005; Mitchell, 2015; Sutton, 2017). Modern university management is focussed on international rankings and encourages entrepreneurialism to secure highly competitive research funding which emphasises competition between institutions (Macfarlane, 2011).

The authority and influence of academics over decision-making within their institutions has been reduced (Bolden et al., 2012; Fredman & Doughney, 2012). The massification of education, increased casualisation of the workforce and greater emphasis on short-term, vocationally driven outcomes (Coates & Goedegebuure, 2012; Ryan et al., 2013) have intensified academic work. Focus on performativity based on ill-conceived research productivity metrics (Kenny, 2017; Kwok, 2013) has resulted in “burnout” and loss of control by academics over their work (Vesty et al., 2018). These changes have challenged the fundamental notions of academic freedom and autonomy (Ball, 2012; Gerber, 2010; Henkel, 2005; Houston et al., 2006; Sutton, 2017).

This paper examines the impact of these tensions on the working lives of academics and proposes strategies to reconcile them, while aiming to retaining the essence of the academic role.

## Literature: managing academic workload and performance

Within universities, workload and performance policies have been developed to manage and control academic work (Boyd, 2014; Burgess et al., 2003; Kenny, 2017; Vardi, 2009). Most universities in Australia have an Enterprise Bargaining Agreement (EBA) which contains a clause dealing with academic workload and stipulates the maximum hours of work which can be allocated to an individual in a given year (NTEU, 2020). However, these clauses do not outline how these hours are to be determined and often vary considerably in their application within a university (Kenny & Fluck, 2014). This has resulted in a proliferation of locally derived workload allocation models which have been largely ineffective at protecting academics from overload (Kenny & Fluck, 2021; Lyons & Ingersoll, 2010).

Lyons and Ingersoll (2010) reported academics as dissatisfied with processes for allocating academic work and the effectiveness of existing industrial agreements to address their concerns. The source of their dissatisfaction is the “seeming reluctance of university managers” to recognise the inherent motivation of academics to contribute to new knowledge in workload models. They further claimed, as the existing policies have led to greater productivity in teaching and research, “the status quo serves the institutional interests of university managers” (p. 138).

While traditionally the autonomy and academic freedom related to academic work make it difficult to quantify and manage, these notions remain “central to an academic identity” (Bolden et al., 2012, p. 36). From the academic perspective, increased productivity in universities has come at a cost. The design of “workload formulae” often limits academics’ ability to do research (Smyth, 2017). More recent research has identified a range of short-comings with existing workload models in universities which focus largely on teaching, but underestimate the time required for many of the associated tasks. They also largely ignore time required for many scholarly activities associated with research and service (Kenny & Fluck, 2017, 2018, 2019, 2021).

For many academics, greater teaching and administrative demands have squeezed time for research, so that much of their research must be done in their “unpaid” time, or they do less (Lyons & Ingersoll, 2010, p. 139). This has led to a loss of autonomy, work overload, stress and mistrust of management (Ball, 2012; Boyd, 2014; Kenny & Fluck, 2021; Sutton, 2017). This is exacerbated by academic performance management processes, which, in most Australian universities, focus on a narrow range of research outcomes and ignore much of the service and other scholarly work academics undertake (Kenny, 2017; Kenny & Fluck, 2021).

This leads to scepticism and anger as academics perceive workload and performance policies as forms of management control (Boyd, 2014) and is “symptomatic of a wider loss of trust within liberal democracies in universities and academic work” (Woelert & Yates, 2014, p. 4).

By contrast, Barrett and Barrett (2008) argued that, far from being a low-level operational issue, academic workload is a key strategic issue and crucial for the effectiveness of a university. They proposed some principles to underpin workload allocation and suggested that effective workload and performance processes require greater collaboration and transparency than is currently the case. Further, these policies need to be linked into other university systems to restore trust between academics and university management.

In summary, the literature above reveals fundamental issues around academic workload and performance have persisted for many years (McInnis, 1999; Houston et al., 2006; Papadopoulos, 2017; Roberts, 2013). More recently, the impact of these issues has been heightened by extra demands placed on academic staff due to the COVID-19 pandemic (Steinþórsdóttir et al., 2021).

This paper reports on the final phase of a longitudinal study which involved the development of a practical tool designed for academics to obtain a more realistic and holistic estimate of the work demands on them. The longer-term aim of this research project is to develop a credible, holistic, transparent and research-based way to manage academic work and performance in the modern university, while also protecting the fundamentally autonomous nature of the work.

## Background to this longitudinal study

Phase one began in 2015 with a nation-wide survey, which received responses from 2526 responses from academics at 40 of the 41 universities in Australia. The extensive quantitative and qualitative data revealed much about the lived-experiences of these academics with respect to workload and performance policies and practices at their universities.

Detailed statistical analysis led to the identification of a wide-ranging suite of activities in which academics engage, each with an evidence-based time value (or allocation). These activities, which relate respectively to the teaching, service and research aspects of their role, have been published previously (Kenny & Fluck, 2017, 2018, 2019).

Phase one ended with a holistic analysis of the data and the development of a set of principles to underpin academic workload and performance polices in universities (Kenny & Fluck, 2021) these principles have been replicated in the Appendix).

## Methodology

This research has followed a grounded theory approach, employing mixed-methods, in which quantitative and qualitative data were triangulated and validated at different stages (Glaser & Strauss, 1967; Strauss & Corbin, 1990). Phase one led us to conclude that, to have any credibility, and to restore trust, academic workload and performance policies need to be designed to capture what academics do across all levels and disciplines. Further, these policies must be based on a clear understanding of the unique nature of academic work and implemented in accordance with the principles outlined in Kenny and Fluck (2021).

This paper reports on phase two of the research, where our goal was to validate these claims. To do this, we used the data from phase one to develop an academic workload estimation tool (AWET), in the form of a spreadsheet. The AWET was designed to enable each participant to select the activities linked to their work, which it then aggregated to provide a holistic estimate of each individuals’ workload over a given year.

Fifty individuals were randomly selected from over one hundred respondents to the original survey, who had indicated their willingness to participate in follow-up activities. This led to the identification of 39 participants for interview, from 21 of the 41 universities in Australia.

In preparation, each participant was provided with a copy of the AWET and the principles. These were used during the semi-structured online interviews, which occurred in 2020. The participants’ names have been replaced by pseudonyms for anonymity.

We discussed their work in detail and how well the AWET was able to capture what they did for the most recent full-year of work, typically 2019. This estimate was compared with their workload according to their institution’s workload model. We also explored the associated institutional processes through their opinions about the principles which had emerged from phase one (Kenny & Fluck, 2021).

## Analysis and discussion

The 25 females and 14 males provided a range of experience levels: 6 were full professors (level E), 5 associate professors (level D), 13 senior lecturers (level C) and 15 lecturers (level B). They represented 11 broad categories of discipline including arts (6), business (1), education (7), science/engineering (4), psychology (2), health and medicine (9), law (1), planning (1), religious studies (1), social science/work (5) and academic administration (2). This variety in workload categories, academic levels and disciplines supports the generalisability of the AWET.

### Technical issues with the AWET

Any usability or technical issues with the AWET, such as activities missing or needing revision, were noted and addressed in subsequent revisions. Some participants identified specific areas where they thought the AWET either under- or over-estimated what they did, but in most cases, these discrepancies were resolved through a conversation. Often the difficulty was associated with a misunderstanding of the terminology used in the AWET to describe certain tasks, or due to the participant’s unfamiliarity with the AWET. This suggests some training in its use would be advisable, as recommended in the principles.

### The effectiveness of AWET as a tool to estimate individual workloads

The AWET facilitated a deep conversation about their work. Many of the participants said they had volunteered to be part of the research because they had complex roles that were not adequately captured within their own work-places. These staff felt this project would help them to explore their work more thoroughly. Six also specifically mentioned that they had kept very detailed records of their workload over several years, so could readily compare the estimates from the AWET with their own data.

#### Workload analyses

The interviews explored how accurately the AWET captured their actual workload, their overall experiences with workload and performance and the extent to which the principles were applied at their institutions.

The participants were categorised according to the proportion of time to be spent on research, teaching and service as follows: teaching intensive, balanced teaching and research, research intensive and academic service/administration (Table 1). It compares their nominal workloads, according to their substantive positions in their institution, with the estimated workload category based on the AWET.

**Table 1:** Summary of nominal and actual workload categories

Category	Proportion of workload time on activity	Nominal workload	Estimated workload (AWET)
Research intensive	50% or more of their time on research-related duties	4	9
Balanced	Approximately equal time on teaching and research, typically about 40%	18	3
Teaching intensive	50% or more of their time on teaching-related duties	16	23
Academic service/administration	50% or more of their time on academic service or administration	1	4
		39	39

The workload categories as revealed by the AWET were different to the nominal workloads. Only 3 academics were shown to be in “balanced” roles compared to 18 nominally. The main reasons for this, as discussed below, related to the fact that many of activities identified in the AWET, linked to teaching, leadership, administrative or coordination roles, were not accounted for in their institutional workload models.

Table 2 shows a summary of the overall workload estimates, based on the AWET, assuming an annual load of 1717 h, over 47 weeks to allow for public holidays and annual leave. The analysis reveals 24 of the participants worked more than 110% of a full-annual workload, while eleven worked more than 150%. The weighted average workload was 132%, or 2252 h per annum which is equivalent to 60 weeks work at 37.5 h per week or working 50 h per week over 47 weeks. This is consistent with previously published data (Kyvik, 2013; Lyons & Ingersoll, 2010; Tight, 2010).

**Table 2:** Summary of overall workloads based on a full workload of 1717 h p.a

Estimated workload	Number	Average weekly hours worked
100–109%	15	38
110–124%	4	43
125–149%	8	50
150–174%	5	59
175–199%	5	68
> 200%	2	95
Weighted average (132%)	39	50.3

The participants believed the AWET provided a more comprehensive and holistic estimation of their work commitments than the workload model used at their institutions:

I do between three to five months over time each year…I know exactly how much I’ve spent on each thing in hours each year. …so, I would say it’s fairly accurate (Sue)

In another example, Larissa thought the 148% estimated for her workload (2534 h) was a very good reflection of what she did:

…every day is ten or twelve hours…It’s more comprehensive than the one we have…I think it captures the hours … the number of hours required to do my job. (Larissa)

Others commented that it highlighted how much of their work was not picked-up by their institutional workload model. Further, that the academic performance management process, which typically used pre-determined criteria based on selective research outputs, bore little relation to the work they actually undertook. The rest of the paper explores these issues in more depth for teaching, service and research.

Please note, in the following teaching discussion, we use the terms “course” and “program”, as both these terms were evident across the sector to describe a recognised bachelor or masters level course, or program. Similarly, corresponding terms “unit” or “subject” were used for the assessable components within these courses or programs.

### Teaching workloads

From Table 1, 34 participants indicated significant teaching duties were expected as part of their substantive role. Of these, 21 provided very detailed summaries of their teaching, and 17 of these (81%) claimed the teaching estimation using the AWET accurately reflected their teaching workload.

Two experienced academics felt the AWET over-estimated their teaching because they needed less time to prepare classes than their less experienced colleagues. Others had difficulty using the AWET to estimate time associated with clinical teaching sessions, laboratory work or work integrated learning activities.

The conversations clarified misunderstandings and enabled adjustments to suit their specific circumstances. For example, four described course (program) coordination roles which included a range of other responsibilities such as intermittent teaching across a range of units (subjects) within the course (program); coordination and involvement in activities such as clinical assessment and capstone assessment; entry/selection interviews for courses and practical assessment activities.

Others commented on activities missing from the AWET, such as time for supervising honours (and masters) students, and time to manage/train sessional staff. The interviews, which were recorded during the COVID pandemic in 2020, also led many to comment on the extra time needed for preparing/adapting material for online teaching, student consultation and webinars in online teaching situations and increased administrative demands.

These conversations also highlighted that many time-consuming but necessary activities related to preparation and planning of teaching were not accounted for in their institutional workload model, which is consistent with Steinþórsdóttir et al. (2021).

Overall, the respondents said that the AWET gave a more accurate picture of their teaching commitments and a chance to identify aspects of their work which would otherwise not be picked-up.

#### Disciplinary differences

The interviews also revealed differences regarding the teaching situations across disciplines, especially where students were involved in practical or clinical contexts. The need for close supervision, related to safety and/or professional accreditation requirements was apparent in health-related disciplines, such as nursing, dentistry and psychology, where students had to be closely supervised and assessed in small groups or individually. This contrasted with more traditional teaching situations in other disciplines, such as lectures and tutorials.

…so…when you’re dealing with medicine, dentistry…it’s actually a clinic with live patients...that’s why I wanted to participate because you don’t actually capture that... (Larissa)

Others commented on the need to mentor sessional staff teaching into their program to maintain standards. Additionally, Cassie and several others highlighted the need to provide pastoral support for students:

I think there has to be that sort of recognition that all parts of your job, if they take time, and they add to the good of the university and good for students… sometimes it’s the pastoral work you do with students, like spending half an hour on the phone which keeps them in the course… (Cassie)

Shannon, who coordinated a work integrated learning (WIL) program, had major responsibility for organising field work and placements for students said a major part of her job was “outward looking, contacting organisations and negotiating placements”. While dealing with external partners relied on her expertise and professional credibility, she claimed it was not acknowledged within the university. She was afraid her managers might misuse a tool such as the AWET to discount much of her work as administrative and allocate her more teaching:

And it’s because of the difficulties of capturing everything that people do…it’s a bit of a double-edged sword …it could be helpful in arguing for more resources, but there’s a potential for universities to use it. …to force people to do more work basically (Shannon)

Shannon’s comments demonstrate the lack of trust alluded to earlier and raise an important point. There is scope for a discussion to clarify the proportion of her role that involves her expertise when interacting with a professional community or liaising with clients as opposed to the administrative aspects. It would be economically sensible to provide administrative support to enable Shannon to focus predominantly on the more professional aspects of the role. Other participants also mentioned increased administrative demands and questioned the economic value of using their time on such tasks.

It was clear these coordination roles involved a significant element of service through peer support of teaching colleagues to facilitate the continuity and smooth operation of a course (program). However, these academics not only felt much of their work went unacknowledged by their institution, but they also felt professionally disadvantaged because the immediacy of these tasks demanded priority over other parts of their role, particularly research:

I wish I was better at prioritising the research part. And part of me feels like I can’t do that sometimes, because of the other things that are more immediate…I mean teaching is immediate. It’s got deadlines, and you’ve got students and things like that. (Cassie)

#### Summary

The design of the AWET enabled the participants to get a more complete and accurate estimation of their teaching workloads due to its ability to capture a wide-range of activities (Kenny & Fluck, 2017). It provided time allowances for coordination, planning and preparation, assessment and consultation. It allowed for different modes of teaching such as online and face-to-face and a range of activities including lectures, tutorials, workshops, laboratory and clinical sessions, etc. Further, it differentiated between course planning and preparation for teaching, including differentiated allowances developing something totally new, a substantial review or a regular update of existing materials.

Coordination roles also linked to service-related activities in the AWET and demonstrated the close relation between service and teaching. Other aspects of service will be explored in the next section.

### Academic service

Freidson (1999) and Boyer (1990) emphasised “service” as central to academic work. Kenny and Fluck (2019) highlighted its importance for the sector and for universities to function, and identified a suite of service-related activities, with associated time allocations, which have been included in the AWET.

During the interviews, participants described a range of service activities, both internal and external to the university, to support their profession, their community, their peers, their discipline and/or their institution. Examples included committee membership, peer-reviewing articles and editing journals, etc.:

So, I am an associate editor of two journals… I reviewed two journal articles. And I reviewed five grant applications… I am the Chair of a research committee… external to the university...I’m also on a board of directors of a not-for-profit organisation… (Matilda)

Five respondents described how service in the community, was integral to maintaining their currency and proficiency in their field and supporting their discipline:

I run women’s health clinics for one of the local GP practices as part of my service role. It’s a practice where university students are placed and it also [serves] to keep up my registration and accreditation as a health care professional… I do two clinics a month. On average that would be 16 hours a month, times 11 months… (Jade)

Others, in leadership roles, mentioned supporting colleagues and up-coming researchers through participation in scholarly peer feedback events such as “confirmations of candidature”, and organising visiting international scholars.

Two participants suggested that university managers value the prestige these activities bring to the university, but they view them as “voluntary” activities rather than a professional obligation. The participants claimed their institutional models typically failed to adequately acknowledge these service aspects of their work, which further undermined the credibility of the models used.

#### Gendered nature of service

Steinþórsdóttir et al. (2021) argued “gender is rarely discussed in the research debate that surrounds the allocation of academic workloads” (p. 1862). They were concerned that workload models, which tend to undervalue many service-related “chores”, are “especially acute for women” (p. 1862).

With 25 females in this sample, these observations were very pertinent. The experiences of these participants indicated that both male and female participants experienced career disadvantages arising from workload allocation and performance practices that discounted teaching and service work. Wider literature suggests, however, women tend to be disproportionately involved in these roles, which, while necessary for the university to function, are considered low status or “low promotability tasks” (Babcock et al., 2017; Guarino & Borden, 2017; Steinþórsdóttir et al., 2021).

IN support of this, four female participants gave detailed descriptions of receiving little recognition for the time required to keeping a course (program) functioning, by supporting colleagues and students and felt this was detrimental to their own careers.

#### Using the AWET to manage service work

While universities relied on these staff to ensure things ran smoothly, much of this work tended to be viewed as voluntary, ignored or devalued and this undermined trust in the workload allocation and performance processes at their institutions.

Newport (2019) was concerned with the encroachment of administrative tasks on academic time, because it distracts from their primary creative functions of teaching and research. He maintained the costs of administration “remain hidden in ways that don’t immediately show up on a university’s balance sheet”. He called for a means to negotiate “trade-offs” so individual academics could specify an agreed “amount of time…to devote to service each year”.

This is not new. McInnis (1999) noted “the strong perception” amongst academics that administration was “an increasing and seriously intrusive burden” (p. 26). He reported the average proportion of worktime that full-time academics spent on service-related tasks, including administration, was between 22% and 25%. This is consistent with workload categories mentioned in Table 1 and suggests a typical service commitment of around 20% would enable academics to fulfil responsibilities to their profession, discipline and/or institution. However, when certain roles involve substantially greater service commitments, individuals in these situations can discuss “trade-offs” against their research and teaching to compensate for this time.

If we accept these tasks need to be done for the sector to function (Kenny & Fluck, 2019), Newport’s arguments can be extended to other aspects of service, which should be acknowledged in individual workloads and properly costed.

#### Summary

The AWET is designed to be used in conversation with individual academics to discuss their service commitments and how this work will be acknowledged, so their performance is judged on what they do. The list of service activities in the AWET enables this in three ways. Firstly, by enabling an academic to specify specific service commitments for accountability purposes during workload planning discussions. Secondly, it also includes allocations for a range of internal and external administrative service roles. Thirdly, it enables individuals in more demanding service roles, once they have a realistic estimate of the service demands on their time, to identify and negotiate additional time to fulfil these roles and discuss “trade-offs” against other aspects of their work, get appropriate administrative support (as in the case of Shannon mentioned above) or forego certain duties.

In addition, this section of the AWET also allocates a blanket 120 hours per annum to all academics as an allowance for attending to email and miscellaneous tasks. The participants thought this was a good inclusion because it acknowledged the notable increase in these demands.

### Research and scholarship

Feelings of frustration and mistrust were expressed by participants who reported being judged as underperforming, largely based on their lack of research output, while much of their actual work effort, although important to the functioning and reputation of the university, went unacknowledged:

When I got frustrated with everything in 2018, I wrote a very sharp memo to my head of department and Head of School to identify that I am doing 80% of my research my own time…my workload has been like this since I started…I didn’t have any opportunity to do [research]. So, if you look at the way…citations and everything goes, I lost any sort of compound effects…in the last couple of years…I just sort of accepted that my research career was gone. (Sue)

Portia reinforced the comments of others about how the underestimation of many demands can consume the time an individual has available for research and scholarship:

[The AWET] …makes it really clear that…a lot of the work of teaching is sort of concealed…and that’s clearly the thing that disrupts the research productivity. I mean, it’s not something that is a mystery, is it? (Portia)

This situation appears to be widespread in Australian universities because time to do research is pushed to the margins or overshadowed completely (Kenny, 2017). This can have a detrimental effect on the welfare and career prospects of academics (Lyons & Ingersoll, 2010). Margaret described how this situation is exacerbated by institutional performance processes that focus on research output, regardless of the other demands on her time:

So…a large percentage of my time is taken up with teaching, [but] all of the performance metrics that I’m measured on are research performance metrics. … ‘How many grants did you get? How many research publications have you got out? How many higher degree research students are you doing?’ And we sit there and I say, ‘Well, I spent a heap of my time teaching the medical students who bring in an awful lot of money to the university. Can you credit any of that, please?’ And they just don’t. (Margaret)

Others reported experiences where their research time was reduced while legitimate scholarly work, linked to improving teaching, presenting at conferences and/or publishing in professional journals was discounted, because they did not fit with output-based metrics used to determine the time an individual will be allocated for research (Kenny, 2017).

I’m supposed to submit, I think it’s two or three articles to category one journals and bring in $20,000 research funds. But that’s what you’re supposed to do no matter what your research allocation is. So, someone on 40% would be expected to do the same as someone on 10%. (Kendall)

These output metrics are typically derived from external research university accountability metrics linked to the Excellence in Research Australia (ERA). Many other countries have similar metrics (Kwok, 2013), which are meant to compare research performance between institutions. Sean, a professor who is usually “working on several books and articles at any given time”, commented on how external funding priorities suddenly changed and impacted institutional research priorities, when the status of publications was downgraded as an indicator of research quality by the government and the focus shifted to competitive research income:

We’ve been told now for the last two years, basically that published research, ‘yes, it’s nice’ but really, the only thing the university is counting is research income. (Sean)

Despite having previous success with two highly competitive Australian Research Council (ARC) discovery grants, Sean knew “how hard they are to get”. He felt this was “becoming a bit of a mug’s game”. Sean’s and Kendall’s experience are consistent with Kenny (2017) who argued that expecting individuals to win highly-competitive grants and/or publication in tier one journals is inappropriate as an indicator of satisfactory work performance or as an accountability mechanism for research time. With failure rates of around 80% (ARC, 2022), such achievements are clearly beyond the norm and something over which individual academics have little control. When used in this way, they set up 4 out of 5 to fail. Consequently, some participants described situations in which their work performance was judged against criteria that bore little or no relationship to the work they did, or over which they had little control:

I see very little connection between the [workload model] form I fill out at the beginning of semester and what I actually do…with research, my understanding is that there’s no breakdown in that whatsoever. It’s all output. (Amanda)

This mis-alignment between workload and performance policies, particularly for researchers with significant teaching and service roles, limits their opportunities to develop and build a research profile and reduces academic freedom and autonomy. Institutional models which link the allocation of research time to limited previous outputs conflate workload and performance and force many individuals to squeeze time for research into their own time (Kenny, 2017; Lyons & Ingersoll, 2010).

When institutions take this path, it is very risky for individual academics, especially when research time is allocated retrospectively based on selective research outputs. Given the failure rates mentioned above, if they invest the time and fail to publish or win a grant, it can lead to a reduction in time for research in their workload. As many of these participants pointed out, this generally means they are allocated more teaching, leading to a downward spiral and eventually disadvantaging them in terms of future research opportunities and/or promotion.

By contrast, Kenny (2017) argued the research workload and research performance should be separated. The separation of research workload from research output is further underscored by an unavoidable fact. Typically, as Daniel indicates, there is a time-lag between scholarly work being done and any subsequent outputs:

…with those journal articles, right? …12 last year…2019…a lot of the work on those was done …in 2018 and 2017... [maybe] probably …half of it in the year I submitted and the other half in the preceding period…And that’s a problem…. a year is kind of an arbitrary unit of time for academics… it just gets done when it gets done. (Daniel)

Further, developing and managing research projects takes time. Ideas have to be discussed, proposals have to be developed and ethics applications have to be approved. If a proposal is successful, there is still considerable work to be done. Chris, a research-intensive academic, kept detailed records of his work on collaborative projects over several years and described the time needed to manage and interact with external project partners:

It’s been left to me to do things like run meetings, write up reports, and all of those sorts of management kind of things… it’s the nature of the research…some [of which] involves multiple partners. So, there’s a lot of work involved in communication… to make sure that they do what’s expected. (Chris)

Additionally, both Chris and Daniel claimed, they had to continuously apply for funding due to the uncertainty of success for any given proposal. This can also create workload problems because:

…you’ve just got to cast out so many lines and see how many nibbles you get with the sort of research that I’m involved with. And that takes time, but also you don’t have a lot of control over when you hit those levels. And they often happen all at once. (Daniel)

Thus, research workload and research performance, while separate are linked (Houston et al., 2006; Kenny & Fluck, 2019; Pink, 2010). Research workload should aim for individual accountability by ensuring time for participation in the necessary scholarly and related activities. Research performance, on the other hand, should be judged on the outcomes of these activities. This approach would enable scholarly work such as reports, conference papers, publication in lower tier journals, etc. to be acknowledged as legitimate scholarly outcomes, along with the more prestigious outcomes. The relative prestige associated with certain outcomes is more appropriate for conversations around awards and promotion.

#### Summary

The experience of these researchers demonstrates a need to prospectively plan for research time in their workload, to undertake the scholarly activities that may subsequently result in research outputs.

The research section of the AWET has been designed to separate research workload from performance. It enables a research workload, be it 20%, 40%, 80% or some other proportion, to be constructed by reference to the comprehensive list of scholarly activities (Kenny & Fluck, 2018). It facilitates prospective conversations about planned research activities, each with an associated time value (allocation). Examples of activities include developing grant proposals, ethics applications, gathering and analysing data, preparing articles for submission, etc. This ensures accountability for research time and enables reasonable expectations to be set. Conversations about research performance can be held later, based on the outcomes of these activities.

### Using the AWET to manage performance

To re-build trust between academics and their managers, the academic performance policy must also be linked to a credible workload allocation process (Barrett & Barrett, 2008; Kenny, 2017). The principles outlined in Kenny and Fluck (2021) also indicate the need for these policies to work together. This raises the question: what might an appropriate academic performance process look like?

Pink (2010) argued judging the performance of those involved in cognitively demanding work is more effective when designed to suit the intrinsic motivators that drive them. Other researchers have called for an “agency-based” approach to managing academic performance (Barrett & Barrett, 2008; Franco-Santos et al., 2014; Houston et al., 2006; Kenny & Fluck, 2018, 2021). Agency-based approaches assume academics have some control over their work and fit well with fundamental notions of academic freedom and autonomy. Unfortunately, agency-based approaches are not evident in Australian universities (Kenny, 2017).

When asked how effectively the institutional model deals with workload issues, Portia described an experience similar to that of others as:

…hugely disappointing. So, you know, instead of a negotiated agreement, in my experience it has more been of an imposition followed by a defensive struggle, followed by capitulation on my part. So, it’s been …too nebulous to be able to use the model …as a negotiating tool. (Portia)

While workload clauses are included in EBAs, we note that academic performance management policies in universities tend to be outside of the industrial agreements (Kenny et al., 2012). We suggest this might explain the mis-alignment between these policies and why, from a legal and industrial point of view, EBA workload clauses have proven to be ineffective in addressing academic concerns over workload (Lyons & Ingersoll, 2010). Cassie’s experience below, which also echoes the experiences of others, illustrates how this might play out in reality:

I bring my workload to my performance management meetings, …I had to make an effort for my performance manager to look at it because they would say, ‘Oh, I’m just looking at your teaching, I’m not looking at the other parts of your workload’. So, it’s this lack of connection between the whole of the work that you do and, and (so) you tend to forget what you do and you just tend to add on bits. (Cassie)

In effect, Cassie’s performance manager wanted only to consider 40% of her workload and ignore the remaining 60% involving her research and administrative duties. Others also mentioned this reluctance of their managers to deal with anything other than teaching and a tendency to delay or avoid addressing broader concerns around workload. Lyons and Ingersoll (2010) argued that the status quo suits managerial priorities. Two other respondents expressed similar views: that the reluctance of managers was because work that was currently being done “voluntarily” by academics would have to be costed:

I think for universities it makes, it makes business sense, right? …Why would you say ‘no’ as a business to that? You just say, ‘Yes, sure! You can work hard for free if you want.’ But I think the consequences of that is no one takes annual leave, and everyone’s burned out. (Daniel)

Similarly, others commented that there was no method of accounting for contingencies due to changes in personal circumstances, such as extra work due to winning a grant during the year, taking on new research students, taking leave or covering for one’s own or a colleague’s sickness. Further, as these interviews occurred in 2020, at the height of the COVID 19 pandemic, this lack of an effective process to adjust their work commitments on a holistic basis was exacerbated by fear of job losses and the extra demands placed on them:

You know, some of our staff especially with COVID are working ten-hour days…they’re absolutely exhausted. And yet on paper …it’s just not reflecting that… it was probably driven by the executive…for the whole university to try and help them…plan their financial planning. (Jade)

When properly aligned, the workload allocation would consider an individual’s whole contribution and their performance would be judged on that basis. Significant changes in their conditions of work would be reflected by corresponding changes to the performance expectations. This fundamental mis-alignment between academic performance management and workload allocation must be addressed. Otherwise, as Papadopoulos (2017) pointed out, these policies are open to manipulation, despite the existence of a workload clause in the EBA:

…[workload] models provide a virtual site wherein major changes to educational practices can be introduced through alterations to models…this opens up the opportunity to drive change through the workload [rather] than by consultation and consideration of impact upon academic workers. (p. 522)

This also points to the need for good management. Some participants mentioned examples of constructive and helpful interactions with managers. Several, including Daniel, described how a supportive head of school had helped him to build his career. From this experience, he argued what should be recognised in conversations with an individual is their contributions. As someone who had been a head of school, Basil emphasised the need for heads to be trained:

…to get these principles through. I think …a strong program for heads of school, particularly new heads of school, to learn how to be a Head of School. Just because you’ve been an academic for 10 years doesn’t mean you know how to do this stuff… not everybody is good at managing staff. (Basil)

Cassie commented on the importance of a supportive leadership in approaching mature professional conversations about workload, in a non-judgemental and non-confrontational way for the process to work. Daniel further commented on how the transparency and objectivity of the AWET would prevent individual academics from inflating their workload or highlight when some “fiercely independent” individuals may be taking on too much. Margaret commented on the potential of the AWET to be used in a conversation about her work to ensure more fairness and equity in allocating workload:

I like this, I actually think a systematic process of thinking through all the elements of the teaching is needed… And I think also that the other thing about something like this tool is that it shows you areas where you could… aspire to… (Margaret)

In accordance with the principles, there needs to be an institutional approach in which the AWET is implemented and resourced. However, in recognition of the inherent power differentials in the modern university and the fear of many academics that this process can be co-opted by managers, implementation and operation should be jointly managed by representatives of both the academic staff and university management.

Drawing on Kenny et al. (2012), and the principles, we recommend the establishment of a workload committee, to oversee the implementation and operation of the institutional policies. This committee would have equal representation from academic staff and management. The academic staff representatives should be nominated by their union. The committee would consider and rule on any proposals for additional allocations to those in the AWET at the discipline level. The allocations in the AWET are research-based, so any proposed changes must be based on credible evidence and voted on and supported by a clear majority of the affected academic staff. Once in place, where individual workload disputes cannot be resolved within a work area, the formal dispute process would be activated.

#### Summary

Our research confirms that academics value the autonomy and flexibility of their role but want their work to be properly acknowledged. While most are reluctant to count hours (Kenny et al., 2012; Papadopoulos, 2017; Steinþórsdóttir et al., 2021), any credible workload estimation process requires some process to capture the work undertaken. It must be sufficiently realistic to enable a meaningful conversation about their work performance.

The AWET does this, not by counting hours, but by documenting the activities related to their teaching, research and service roles. Each activity has a credible and evidence-based time value (allocation) attached to it. When these activities are aggregated using the AWET, it provides a more transparent, holistic and realistic estimate of an individual’s workload.

As these time allocations are the result of a statistical averaging process, they do not suggest that any individual can be expected to complete a given task in the allocated time, so they must not be used as a basis for “time and motion” studies. The aggregated times can be used, however, for comparison purposes to ensure equity and fairness and to inform a conversation about the development of performance expectations related to an individual’s contribution.

## Conclusions: implementing the AWET

The second phase of our research aimed to validate the findings from phase one. We developed the AWET based on those findings and used it in interviews with 39 academics to discuss in detail their workload for a recent year. For each participant, the estimate from the AWET was compared with the estimate using their institutional workload model. We also discussed their lived experiences with institutional processes concerning their workload and performance and the principles put forward by Kenny and Fluck (2021).

These conversations revealed the diverse and complex nature of academic work and a general loss of autonomy and control by academics over their work. The findings confirmed that often an individual’s performance is judged against criteria unrelated to major aspects of their work, so many felt disadvantaged because the workload process disregarded or undervalued much of what they did. This was especially evident where the immediacy of teaching and service demands took time away from research. The interviews confirmed the general lack of trust due to the mis-alignment of workload and performance policies which do not genuinely attempt to capture what they do.

The AWET was designed to address these persistent issues with academic workload models in universities. As it is based on research and was derived and validated against the lived experience of academics from across Australia, we offer it as a universal and standardised method for individual academics to capture their work commitments in a holistic and transparent way.

The AWET should empower academics in several ways: firstly, by providing a comprehensive list of activities, each with pre-defined and validated workload value (allocation), to obtain a credible estimate of what they do; secondly, by enabling a conversation with their performance manager to negotiate a reasonable workload for the up-coming year and to identify trade-offs as required; thirdly, by helping to prospectively plan their career path and research trajectory; and fourthly, the figures in the AWET cannot be changed unilaterally by managers to suit a budget.

Benjamin (2010) suggests that to be legally binding, the use of the AWET and the principles need to be embedded within university EBAs. Our strong recommendation, therefore, is that the AWET be adopted as a standard model across the sector. We call on university management and the union to take up this challenge and work together to finally get this right as a key strategic process to benefit academics and therefore, the effectiveness of universities (Barrett & Barrett, 2008; Lyons & Ingersoll, 2010).

When implemented by an institution, in accordance with the principles outlined in Kenny and Fluck (2021), the AWET should enable academics to get a more reasonable match between expected performance and the work undertaken. It should help to restore trust between academic and managerial staff in universities, help to build a common purpose and improve university effectiveness.

We highly recommend initial training for institutions intending to adopt the AWET. We encourage individuals or institutions to access it, use it and provide us with feedback. In accordance with our ethics approval, any data collected through this feedback process will be treated confidentially and used to improve and develop the AWET over time.

To facilitate the advancement of this work, and for any readers wishing to try the AWET to estimate their own workload, it has been made available, along with user documentation, ethics information and a link to an online survey for feedback. To access this website click on or copy the following link into your browser https://www.awet.edu.au.

## Appendix 1 Principles underpinning academic work

The policies related to academic work in a university need to be trustworthy. At the institution level, this will be achieved when:

1. They are developed, documented and implemented in full consultation with academic staff.
2. They are based on an acceptance of the intrinsically motivated and self-managed approach that academics are expected to take to their work.
3. They are adequately resourced, adopted across the institution and directly linked to other institutional processes such as budget and performance management.
4. The associated processes and tools are clearly visible and readily available to academic staff and their performance managers, to facilitate genuine negotiation about career goals, workload and performance expectations.
5. All staff and managers receive training in the application of the policies as required.

The outcomes for individual academics will be trustworthy when these policies:

6. Are applied in a fully transparent manner, in terms of process and outcome.
7. Provide a holistic estimate of an individual’s workload, based on realistic time allocations for all key tasks they are expected to undertake in their teaching, research and administrative/service roles.
8. Are sufficiently flexible to cater for justifiable variations associated with differences in discipline, career stage and workload category.
9. Enable individual academics to negotiate reasonable workload and performance expectations that mirror their agreed work commitments.

From Kenny and Fluck (2022)
